# Reasons for sleeping difficulties as perceived by adolescents: a content analysis

**DOI:** 10.1111/scs.12750

**Published:** 2019-09-05

**Authors:** Malin Jakobsson, Karin Josefsson, Karin Högberg

**Affiliations:** ^1^ Faculty of Caring Science, Work Life and Social Welfare University of Borås Borås Sweden

**Keywords:** sleeping difficulties, adolescent, perception, questionnaire, content analysis

## Abstract

Sleeping difficulties are increasingly prevalent among adolescents worldwide and have negative consequences for adolescent health and education. The aim of this study was to describe the reasons for sleeping difficulties as perceived by adolescents. Sleeping difficulties include insufficient sleep, trouble falling asleep, waking up at night or sleep that does not leave an individual rested. Data were collected in 2015 using an open‐ended question. The sample consisted of *n *= 475 adolescents from a city in Sweden, aged 15–16 years, with self‐assessed sleeping difficulties. The results described the reasons for the adolescents’ sleeping difficulties, at a general, thematic level, as an imbalance between requirements and preconditions, distributed to stress, technology use, poor sleep habits, existential thoughts, needs and suffering. To find a balance in their daily lives, adolescents may need support from parents, school nurses and school health services to deal with their sleeping difficulties.

## Introduction

Sleeping difficulties with insufficient sleep are increasingly prevalent among adolescents worldwide 1, 2 and are an international public health issue 3. As a term, sleeping difficulties covers problems related to insufficient sleep 2. In this study, the term sleeping difficulties are used to represent insufficient sleep, including trouble falling asleep, waking up at night or sleep that leaves an individual unrested. The sleep recommendation for adolescents is eight to ten hours each night 4. According to Matricciani, Olds and Petkov 5, adolescents’ average sleep duration has decreased by more than one hour over the last 100 years. Norell‐Clarke and Hagquist 6 also showed decreased sleep duration over time: in 1985 one in ten adolescents went to bed after 11:00 pm, while in 2014, six in ten adolescents went to bed after 11:00 pm.

Adolescents with sleeping difficulties are at risk since there are associations with reduced learning ability, memory impairment, hyperactivity, worsened school performance, lower grades, and increased risk of depression and anxiety 7, 8, 9. Factors that negatively affect sleep are loneliness 10, negative family environment, technology use (other than television), evening light, presleep worry, and the use of caffeine, tobacco and alcohol 11. Adams et al. 12 showed that socialising, fear of missing out, and social and technological distractions negatively impact sleep during the first semester of college. There are also factors that positively affect sleep, such as positive peer relationships 13, good family environments, parent limit‐setting, good sleep hygiene and physical activity 11. Technology use at bedtime, strong emotions and sports participation are both barriers to and facilitators of healthy sleep 14.

Adolescents’ sleep habits may also be influenced by being in a developmental phase characterised by changes and existential thoughts. According to Erikson's 15 development theory, the phase between 12 and 20 years old entails identity confusion and a search for sexual identity, interests and career choices. This phase is a period in which adolescents face making different choices, experiencing feelings of a fragmented existence, and, sometimes, an excessive adjustment to the will of others. This maturation process also includes the progress of abstract thinking and influences the adolescent's view of the concept of health 16. This phase also offers opportunities to shape personal capabilities and routines for later life. According to investigations on the public health of adolescents 17, 18, the phase of adolescence is well‐suited for building enduring habits. Health habits consolidated during adolescence are often kept in the future.

Sleep habits play a crucial role in healthy development among adolescents and have a strong and specific relationship to adolescents’ learning capacity 1, 7, 19. Health is an important goal in school health services that can be pursued by creating environments that promote adolescents’ learning, development and health. Promoting health includes health both at school and from a life perspective 20, 21.

Altogether, the current state of knowledge about adolescents’ sleeping difficulties is mainly based on questionnaire studies with fixed response options or other quantitative data. It is important to describe adolescents’ own perceptions of health problems to ensure the necessary adequate evidence for preventive care interventions 18. Therefore, it is necessary to study the reasons for sleeping difficulties as perceived by adolescents.

## Aim

The aim was to describe reasons for sleeping difficulties as perceived by adolescents.

## Method

### Design

**Figure 1:**
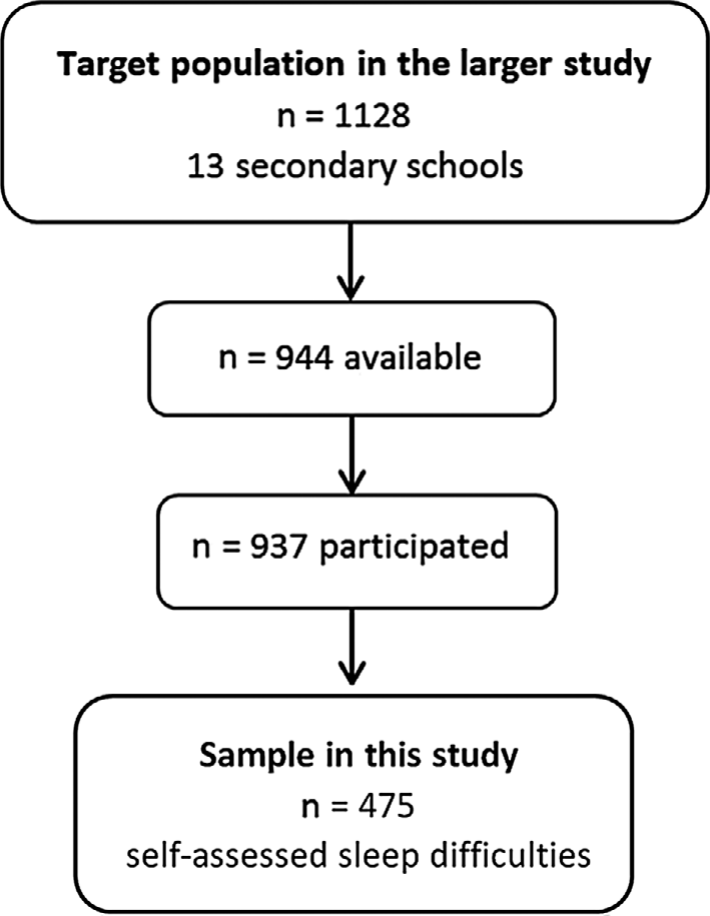
Flow chart of participants.

The design was descriptive, with a survey research approach with an open‐ended question 22. The study was a part of a larger questionnaire survey performed May–June 2015 in a city of Sweden 23. The larger study described adolescents’ self‐reported sleep duration and sleeping difficulties, and explored their associations with school stress, self‐perception and technology use 23. Of the *n*= 937 adolescents at 13 schools in the larger study, *n* = 475 expressed self‐assessed sleeping difficulties and comprise the sample for this study (Fig. 1). Data were analysed using qualitative content analysis 24, 25 and quantitative content analysis 26. The study was approved by the Ethical Board.

### Participants

The participants consisted of *n* = 475 adolescents from all 13 secondary schools in a Swedish city with approximately 110 000 residents. Nine of the schools were in the municipal sector and four in the private sector. The municipalities consisted of rural and urban areas socioeconomically similar to the national average. Together, the 13 schools had *n* = 1128 adolescents (15–16 years old) in the ninth grade, which is the last year of secondary school for this age group before applying to three years of upper secondary education.

The adolescents in this study (*n* =  475) consisted of 200 boys and 275 girls, and 139 lived with noncohabiting parents and 63 were born abroad. The average grade among the adolescents was 240 credits, slightly higher to the national average in Sweden (m* *=* *225 credits, min–max = 0–340). The average sleep duration was 7.11 hours per night, with a range from three hours to 12 hours per night.

### Procedure and data collection

In the larger study, *n* = 937 adolescents answered a questionnaire with the following open‐ended question: ‘If you perceive that you sleep too little, have trouble falling asleep, wake up at night or that sleep does not make you rest, please write what you think your sleeping difficulties may be due to’. Of these *n* = 937 adolescents, *n* = 475 expressed self‐assessed sleeping difficulties and constitute the sample in this study (Fig. 1).

To collect data, the first author contacted the principals at all 13 schools and informed them about the study. Thereafter, the principals received the same information in written form, and information was also sent to the adolescents’ parents. All the principals gave their permission to conduct the study, and they provided the researchers with the names of teachers. The teachers were informed about the study via e‐mail, and they suggested times for the researchers to visit the school. One or two of the authors were present in the classroom, and the adolescents were informed about the study verbally and in writing before they completed the questionnaire. The authors were careful not to provide information about the importance of sleep for health, so as not to affect the responses of the adolescents. The entire questionnaire, with the open‐ended question, was completed in ten minutes. The adolescents placed their answered questionnaires in a collection envelope.

### Data analysis

The data consisted of adolescents’ answers, from a few words up to six sentences. They were analysed using qualitative content analysis 24, 25 and quantitative content analysis 26. Qualitative content analysis has an inductive approach; in other words, the categories are not determined in advance but emerge through the analysis. The analysis started by repeatedly reading the data in their entirety. Thereafter, the analysis progressed from meaning units through condensed meaning units, codes and 22 subcategories to six main categories – in other words, an abstraction and interpretation of the text while preserving the core – and finally, an interpretation was done on one theme. Table 1 provides an example of the data analysis. To reach trustworthiness, the analysis was thoroughly discussed in the research group until consensus was reached, and the result was discussed with research colleagues in seminars. A quantitative content analysis was used to count each occasion the subcategories and main categories was stated – in other words, the reasons for sleeping difficulties – to describe their frequencies 26. Table 2 shows an overview of the results.

**Table 1:** An example of the qualitative content analysis

Meaning unit	Condensed meaning unit	Codes	Sub‐categories	Main categories	Theme
The only thing I know is that my sleep does not make me rested, it does not matter how much I sleep; I am always just as tired. I think it may be because I sit too much with the cell phone, etc., and that I think too much about things, always a lot in the head.	My sleep does not make me rested, no matter how much I sleep; I am always just as tired. Sitting too much with the mobile, thinking too much about things, always a lot in the head.	Not rested‐despite sleep.	Tired despite good sleep.	Poor sleep habits.	Imbalance between requirements and preconditions.
		Sitting with the mobile.	Mobile, computer or tablet use.	Technology use.	
		Thinking too much.	Thinking a lot.	Existential thoughts.	
		Always a lot in the head.			

**Table 2:** Description of reasons for adolescents’ (*n *=* *475) sleeping difficulties

Theme	Imbalance between requirements and preconditions					
Main categories	Stress (251)	Technology use (161)	Poor sleep habits (152)	Existential thoughts (85)	Needs (51)	Suffering (35)
Sub‐categories	School stress (151)	Mobile, computer or tablet (137)	Sleeping after school (14)	Concern (26)	Need for food and fluids (16)	Depression or anxiety (14)
	Everyday stress (82)	Movie, series, or TV (14)	Staying up late (69)	Thinking a lot (53)	Natural needs (8)	Bullied (2)
	Do not want to ‘miss out on life’ (18)	Gaming (10)	Unfavourable circadian rhythms (6)	Nightmares (6)	Need for physical activity (7)	Mental illness within the family (6)
			Tired despite a good sleep (47)		Need for a disturbance‐free bedroom environment (20)	Pain or illness (13)
			Lacking routine (16)			

### Ethical considerations

Ethical research principles were followed carefully by fulfilling the requirements of information, consent, confidentiality and usage 27, 28. The principals at all schools gave their informed consent to the study. The adolescents were given written and oral information about the study, including the possible risks and benefits of participating, and were informed that they were free to withdraw their participation at any time without providing reasons. All adolescents were 15–16 years of age. All adolescents gave their informed consent to the study. They were guaranteed confidentiality and anonymity in the manuscript. If any adolescent needed to talk to an adult after participating the study, the parents, teachers and school health services were informed. The adolescents and parents had the contact information of the researchers.

## Results

The results described the reason for adolescents’ sleeping difficulties as an imbalance between requirements and preconditions, distributed between stress, technology use, poor sleep habits, existential thoughts, needs and suffering, with 22 subcategories (Table 2). Table 2 provides the frequencies of the reasons (*n* = 735) for sleeping difficulties. On average, each adolescent identified 1.5 reasons (min–max = 1–6).

### Imbalance between requirements and preconditions

The adolescents’ reasons for sleeping difficulties can, at a general, thematic level, be understood as an imbalance between requirements and preconditions. Requirements were, for example about performing at school, having activities, being social both digitally and in real life, always having fun, settling in on time and fitting in. Preconditions were about having time, structure, parental support and an ability to set their own limits, for example with mobile use or how different social ideals were allowed to influence them. When the preconditions were not sufficient to pair with the requirements, an imbalance arose that interfered with the sleep of the adolescents.

### Stress

The adolescents’ sleep was perceived to be disturbed by stress. Stress included school stress, everyday stress and not wanting to ‘miss out on life’. Stress resulted from both the adolescents’ own demands and external expectations but also the desire to make fun things. Stress was the reason most commonly mentioned reason (251) for causing sleeping difficulties.

School stress was described as a sense of concern, constant pressure and stress about school or schoolwork. It was based on the expectations and demands adolescents placed on themselves. In addition, there were external demands or expectations from parents, teachers or society. School stress came from feeling peer pressure and expectations that could not be lived up to, including stress over homework, exams, submissions and reports. Doing homework and preparing exams and assignments took a lot of time after school and sometimes led to late evenings and nights. In addition to the time aspect, homework induced an internal concern with feelings of inadequacy. During periods of national tests, the stress level increased. The stress in connection with national tests arose from the fact that regular examinations were ongoing at the same time and that the national tests were considered particularly important for the future. Getting good grades was important and created recurring thoughts about the future and the need to get certain grades for eligibility at the desired high school. Stressful and pressing thoughts about grades, homework and school influenced adolescents’ ability to get to rest and fall asleep.

‘…I usually find it difficult to sleep because of school. I have terrible performance anxiety and suffer from it every day…’ (P131)

Everyday stress was described as a feeling of not being enough. Everyday stress refers to things that do not have to do with school or schoolwork. Everyday stress was associated with the attempt to live up to everything the adolescents themselves wanted, as well as what they should do or be. What should be done was shaped not only by their own requirements and the expectations of their family and friends but also by the ideals of society. This was expressed as a desire to help at home, spend time with loved ones and hang out with their friends, while at the same time feeling there was not enough time. Society's ideals included, among other things, beauty ideals and the need to follow trends and always be stylish. In some cases, exercise caused stress through late evening training that caused difficulties in unwinding and getting into bed early. Long journeys to the training facility, early mornings and pressure from the coach or club led to a reduced priority for homework and sleep. Everyday stress led to a feeling of not being rested after a night's sleep, as the stress made it difficult to relax.

‘…I am constantly stressed, sleep very badly, and I am never fully rested. Soon I break down. There are no improvements…’ (P15)

Stress also affected those who do not sleep because they did not want to ‘miss out on life’. These adolescents did not perceive sleep as fun. They perceived that there were more enjoyable things to do and that they might have missed something while they slept. They also thought it was weak to sleep, and that sleep was overrated. Despite a few hours of sleep, sometimes only four hours, these adolescents had a sense of well‐being. They prioritised living life to the fullest oversleep.

‘…because I live life and then you do not have time to sleep…’ (P261)

### Technology Use

The adolescents’ sleep was also disturbed by their use of technology, that is mobiles, computers or tablets; movies, series or TV; and gaming. Using technology was either something the adolescents wanted to do, something that ‘should or must’ be done or something that was difficult to refrain from. The use of technology was considered the second most common reason for sleeping difficulties (161).

Mobiles, computers and tablets are used for surfing, watching interesting things and being on social media, such as Instagram, Snapchat and Facebook. There was a perception among the adolescents that others expected them to be available around the clock, which led to difficulties in shutting down the technology. Having the mobile switched on meant the adolescents risked being woken by notices or audio signals. Along with the sounds, the knowledge that the mobile was lying alongside the bed and that something could ‘happen’ caused sleep disturbances. Mobiles, computers and tablets are also used for homework, information searches and entertainment. Some adolescents felt that the frequent use of a computer or tablet in school and for homework after school led to a technology addiction that was not always self‐chosen. It was considered difficult to get rid of the mobile, computer or tablet, which delayed bedtime.

‘…I think it can be difficult to sleep because you have to be social on social media…’ (P94)

The use of technology in the form of watching movies, series or TV was perceived as relaxation, which also delayed bedtime since the activities felt difficult to interrupt. Watching movies, series or TV was considered more fun than sleeping and thinking about school the next day. There was a duality in watching movies or series at bedtime. On one hand, adolescents expressed that sleep was delayed, but on the other hand, it could facilitate sleep and thereby alleviate sleep problems.

‘…I think my sleep problems are because I do not go to bed because I stay up and watch TV series until late at night…’ (P320)

Gaming was about wanting to play different computer or video games. This caused late evenings and nights, which delayed bedtime. Late gaming required a lot of concentration in the last waking hours, which meant the adolescents did not relax before it was time to sleep. Gaming was described by some as an abuse they could not escape, thus preventing them from getting enough sleep.

‘…it may be because I am addicted to gaming…’ (P38)

### Poor sleep habits

The adolescents’ sleep was disturbed by poor sleep habits, including sleeping after school, staying up late, unfavourable circadian rhythms, lacking routine and tired despite good sleep. These poor sleep habits were not related to any specific external cause; the adolescents only reported facts. That the adolescents’ sleep was disturbed by poor sleep habits was stated on 152 occasions, meaning it was the third most common reason for sleeping difficulties.

Sleeping after school meant that night sleep was disturbed. After‐school leisure was switched by some to a sleep‐time that lasted from half an hour to several hours. Sleeping after school led to a negative spiral as it became difficult to fall asleep in the evening, resulting in a shorter night's sleep, tiredness during the day and a need to sleep after school once again.

‘…I get so exhausted after school that I sleep 2–4 hours. Then I have a hard time getting a good sleep, which means that I get tired the next day…’ (P184)

To stay up late led to a shortened night's sleep. Staying up late did not depend on time pressures; rather, it became a habit. The leisure time between school and bedtime was devoted to, among other things, homework, technology use, social activities and training, and then it was good to have set time to unwind before sleep. Here, to unwind means to end their technology use, avoid doing homework at late hours, be at home for a while after activities or read a book before it is time to sleep. In addition to the fact that the available sleep hours were reduced, a late bedtime sometimes caused difficulties in falling asleep.

…I sleep too little and do not get rested. Because I am not tired when I go to bed—stay up too long—not rested…’ (P249)

Circadian rhythms disturbed the number of sleep hours for the adolescents. This was especially noticeable for night people, as their biological fatigue happened too late to get enough sleep. Some adolescents understood being a morning, evening or night person as hereditary.

‘…mom and my family … we can sleep about 3–4 hours a night but still be alert…’ (P245)

The adolescents’ sleep was affected by a lack of routine. Lacking routine meant the body did not get any regularity in sleep. Irregularity and a lack of routine were not taken seriously by the adolescents, who were indifferent to them. They also expressed that it was difficult to achieve good routines if they were previously missing.

‘…I have not created any routines regarding sleep; therefore, I often reverse day and night and stay up…’ (P228)

Some adolescents indicated they were tired despite having a good sleep. Despite sleeping up to 10–12 hours a night, they were fatigued and usually unaware why. The adolescents felt more fatigue and connected it with puberty and increased hormones. They did not feel ready to get up in time to arrive at school punctually. Some adolescents connected sleeping well and for enough hours but still being tired with experiencing stressful days.

…I fall asleep early, never wake up at night, but still I am dead in the morning. Something seriously wrong with me… (P174)

### Existential thoughts

The adolescents’ sleep was disturbed by existential thoughts, which included concern, thinking a lot and nightmares. *Existential* thoughts were not about school but more about life, relationships, days that had come and gone, and the future. These thoughts felt important but were perceived as constantly returning and disturbing sleep. Having existential thoughts that interfered with sleep was reported by the adolescents on 85 occasions.

Concern meant concern for themselves and those in their immediate surroundings, but also concern regarding bigger issues, such as the horrors that happen in society and in the world. Some adolescents’ concerns led to difficulties in falling asleep. They stated that they needed a safe person next to them at night or that they were having trouble falling asleep when it was quiet in the room. Concerns about their own friendship relationships, love relationships and family relationships also caused some adolescents to wake up at night and be concerned.

‘…I worry too much, so I find it hard to fall asleep and used to wake up several times a night…’ (P303)

Adolescents perceived that they thought too much sometimes. Recurring thoughts occupied their focus, which led to difficulties getting to rest. Some stated that the thoughts never ran out. The thoughts consisted of everything from deeper philosophical thoughts about the meaning of life to reflections on whether they were good as they are. The thoughts led the adolescents to be sad, over‐analytical or stuck in negative thinking. These thoughts happened especially at bedtime but also during the night.

‘…it is like voices in my head that make me cry. Sometimes I get no hours of sleep because I can never find peace. I am never satisfied with myself, nor are my parents and my friends…’ (P380)

Nightmares were a part of existential thoughts. Existential thoughts that filled the adolescents during the day were processed at night and then often appeared in nightmares.

‘…I don't sleep that much because I'm waking up at night and dream a lot of nightmares of things I feel bad about…’ (P251)

### Needs

Adolescents described their sleep as disturbed by different needs on 51 occasions. Needs included the need for food and fluid, natural needs, the need for physical activity and the need for a disturbance‐free bedroom environment.

Adolescents believed that the need for food and fluid should be in balance for optimal sleep. Food and fluid intake at bedtime were perceived as contributing to difficulty falling asleep. But the adolescents also felt that slight feelings of hunger or no food and fluid intake could lead to sleeping difficulties. The adolescents noted that computer games late at night led to the ingestion of food and fluid, such as energy drinks, which provided additional difficulties in falling asleep.

‘…if you eat too much food during the evening it will be difficult to fall asleep…’ (P241)

In some cases, natural needs, in the form of having to urinate, defecate or masturbate, interfered with sleep or caused waking at night. The need to go to the toilet, every night or several times each night, was reported on a few occasions. This need sometimes coincided with late food and fluid intake, but most often, the adolescents stated it was a habit or an undefined need.

‘…then I almost always have to poop at night like at 2…’ (P103)

Unmet physical activity needs disturbed the sleep of adolescents. This meant the body did not feel tired when it was time to sleep. The physical activity the adolescents described involved organised training or exercise and easier movement on their own. If the need for physical activity was met not too late in the evening, a good sleep was facilitated.

‘…you do not exercise as much during the day and then you have unused energy that keeps you awake…’ (P424)

A disturbance‐free bedroom environment contained an uncomfortable bed and a bedroom that was too bright, too hot or too cold. In addition, a disturbing bedroom environment contained pets that barked, bit, or wanted to sleep in bed and parents or siblings who disturbed the bedroom by talking loudly, snoring or waking the adolescents. The lack of a disturbance‐free bedroom environment made it difficult to fall asleep and affected the night's sleep by leading to repeated awakening.

‘…it may be because now that my mother is working in the evening my little brother can't sleep alone, so that in the middle of the night my dad wakes me up at 3 am and I have to sleep with my brother all the time now…’ (P156)

### Suffering

The adolescents’ sleep was disturbed by suffering. The adolescents who suffered included those with depression or anxiety, and those who were bullied, had mental illness within the family, or had pain or illness. In some cases, the adolescents who suffered mentally or physically had contact with care; in other cases, the suffering was something they struggled with themselves. On 35 occasions, it was stated that sleep was disturbed by suffering.

The adolescents with depression and anxiety were affected around the clock. They had anxiety‐filled thoughts that never ceased or a feeling of being on the verge of breaking down. In some cases, the anxiety was related to the adolescent commuting between eating and not eating, which, in turn, affected the body's ability to sleep well. Depression and anxiety caused difficulty in falling asleep, not feeling rested after sleep, or an inability to get up in the morning. For some adolescents, sleeping difficulties had become chronic sleep problems.

‘…I sleep too much. I can sleep for 12 hours. I also find it hard to get out of bed because of anxiety and/or depression…’ (P354)

A part of suffering was being bullied or offended by students in school or via social media in their leisure time.

‘…school violations by other students…’ (P307)

The adolescents were not just concerned with their own health and well‐being. Worrying about other family members’ mental illnesses also caused sleep disturbances. These concerns included family members who were in and out of hospital, family members having a hard time and negative family events affecting the entire family's future mood. Parents’ mental illnesses sometimes led to a parent no longer being able to care for their adolescent. To have a family member with mental illness sometimes caused anxiety and sleeping difficulties.

‘…my mother is eating herself to death, I know she wants to kill herself…’ (P352)

Pain or illness affected sleep every night or every now and then. Pain or illness included, for example problems with the heart and lungs, polyps, allergies, restless leg syndrome, tinnitus, headaches, menstrual cramps, pains and pain after injury. Pain or concern over illness led to difficulties falling asleep or awakening during the night.

‘…my injury in the knee. Because I didn't get the care I need. I often wake up at night because of the pain…’ (P29)

## Discussion

The study's results revealed that adolescents perceived a variety of reasons for their sleeping difficulties: stress, technology use, poor sleep habits, existential thoughts, needs and suffering (Table 2).

The results showed that stress was expressed to a large extent and of a varying nature. This is in line with a worldwide survey by the World Health Organization 29 that pointed out how stress is an increasing reality for adolescents. In a Swedish study, one in three adolescents experienced symptoms of chronic stress 30. Anniko, Boersma and Tillfors 31 showed that school performance is the primary reason for stress, followed by social stress, among adolescents. Yan, Lin, Su and Liu 32 pointed out that academic stress is related to a reduction in sleep quality among adolescents. These studies confirm the adolescents’ perceptions in this study that stress is about feelings of inadequacy related to school performance, being good enough and ‘keeping up with’ others.

Research has focused on how to encourage adolescents to cope with stress cognitively 33 or reduce stress via physical activity 34 or mindfulness 35. However, the design of interventions to reduce stress in school should also be of interest. For example, if stress and sleeping difficulties decrease when adolescents, teachers and principals regularly work and plan examination schedules and other requirements together, during the school term. Gruber, Somerville, Bergmame, Fontil and Paquin 36 evaluated health‐promoting sleep interventions in relation to the school. They revealed significant results from a school‐based sleep education program where school students, parents, school management and decision‐makers received education included the barriers to proper sleep, good sleep habits, consequences of poor sleep and the importance of sleep as a critical part of a healthy lifestyle. The education resulted in extended sleep duration and improved academic performance among the participants 36.

Aside from school stress, another form of stress expressed in this study was the perception of not wanting to ‘miss out on life’. This stress may be related to the concept of fear of missing out (FoMO), which is the pervasive apprehension that others might be engaged in rewarding experiences from which one is absent 37. FoMO is linked to daily life and is specifically related to the use of social media.

Using mobiles, computers and tablets was the second most mentioned reason for sleeping difficulties. The adolescents described their technology use as having to be connected around the clock, making it difficult to stop technology use. Technology use was a common reason for sleeping difficulties in this study which is in line with research by Hale and Guan 38 and Garmy and Ward 39, which determined the links between technology use and sleeping difficulties in the form of delayed timing and shortened sleep duration. Perhaps the adolescents’ technology use in this study was also present in the categories of stress, poor sleep habits and existential thoughts, even though the adolescents did not express this. Since technology use has become a natural part of adolescent life, they might not think about it as something leading to late nights, concern and stress. This reflection is confirmed by the report by the Young Health Movement and the Royal Society for Public Health 40 describing the downside of social media use. The report stated that many adolescents end up in a vicious circle consisting of lack of sleep, tiredness, difficulty coping with life, low self‐esteem and feelings of worry and stress. The report indicated that media use plays an important role in adolescents’ feelings regarding their sleep and well‐being. Additionally, Beyens, Frison and Eggermont 41 demonstrated that FoMO, in the form of an increased need to belong and to be popular, is associated with an increased use of social media. Health promotion work to reduce sleeping difficulties caused by technology use might be complex. Bartel, Scheeren and Gradisar 42 restricted adolescents’ prebed mobile phone use with one hour before bed on school nights, and it led to turning the lights off earlier and sleeping longer. However, adolescent recruitment for that study was low, indicating that adolescents lacked motivation for negotiating changes to their evening phone use. According to Bartel, Scheeren and Gradisar 42 motivating interviews or other cognitive strategies are required to decrease adolescents’ mobile phone use. School nurses and professionals in school health services must lead these important dialogues, as they occupy a unique position in the adolescents’ everyday life.

The third most frequent reason for sleeping difficulties was poor sleep habits, referring to behaviour both beyond and within the adolescents’ control. Beyond their control is the change in circadian rhythms during puberty, which often results in the adolescents becoming alert in the evening and tired in the morning 43. The adolescents’ shift towards an evening circadian preference may indicate the necessity for delayed school start times. Having a delayed school start time has resulted in positive effects on sleep and academic performance among adolescents 44. However, Marx et al. 45 indicated that research could not determine the effects of later school start times because the evidence base was limited.

The adolescents in this study perceived their poor sleep habits as primarily due to circumstances, such as settling down late, not relaxing or lack of routine. Good sleep hygiene habits are fundamental factors for achieving good sleep 11. Research showed that parents’ own routines, parent‐set bedtimes and good family environments are key factors in creating sustainable sleep habits for adolescents 10, 11. Thus, the role of parents is vital. Further, it seems that the adolescents’ perceived reasons for sleeping difficulties stem from lifestyles influenced by their life context and the social norms of society, expressed via stress, use of technology and social media, and lack of routine. Although the role of school nurses includes health promotion, this lifestyle issues cannot be solely their responsibility. Instead, different perspectives are needed when working with lifestyles and norms, such as the neighbourhood, community and decision‐makers.

One reason for sleeping difficulties unrelated to lifestyle was having existential thoughts. Recurring thoughts about life, relationships and the future are common in adolescence. This result was confirmed by Bartel, Gradisar and Williamson 11, who reported that presleep worry likely negatively impacted sleep. It is common for adolescents’ thoughts to turn to rumination, according to Cox, Ebesutani and Olatunji 46, who reported a link between sleep disturbance and repetitive thoughts, such as worry. This result confirms the importance of school nurses being available to listen to adolescents’ concerns and encourage dialogue about existential thoughts even if the adolescents do not speak spontaneously about them. At the same time, according to developmental psychology, existential thoughts are part of adolescents’ natural development 15.

Sleeping difficulties are a health issue related to adolescents’ ability to engage in school education and, therefore, fall within the remit of school health services. Part of the health education provided by school nurses should include a central element of health promotion. This promotion work can take place individually or in groups and can involve classes or meetings with parents 20, 21. This study showed that school stress, everyday stress, technology use and poor sleep habits were the most common reasons for sleeping difficulties as perceived by adolescents. According to *The Lancet* commission of adolescent health 18, adolescence is the perfect time for health promotion work about lifestyle issues because enduring habits may be formed during this time. Further, adolescence is a sensitive time for social learning through behaviour imitation, especially of peers. This suggests that health promotion work concerning sleeping difficulties and their perceived reasons are suitable for groups of adolescents. School nurses should consider adding health promotion work in dialogue with adolescents at a group level along with the statutory individual health visits. As health habits are often established within the family, health promotion work should also include a dialogue with parents, as in parent meetings. According to Baker, Morawska and Mitchell 47, parents play a crucial role in the development of health‐promoting behaviours in both the short and long term. However, parents may need support or formal instruction about how to establish healthy habits.

Additionally, when working on health promotion at the group level regarding the reasons for sleeping difficulties mentioned in this study, it is important that school nurses do not neglect that reasons vary between individuals. School nurses must enquire about adolescents’ existential thoughts, experiences of mental illness, needs and suffering. By caring and genuinely listening when carrying out care interventions to inspire the adolescents to good basic sleep habits and increased self‐control, school nurses and school health service can reduce adolescents’ sleeping difficulties.

### Limitations

This study has some limitations. One might be that the questionnaire with the open‐ended question was answered during 10 minutes. This might imply a limit on the adolescents’ expression, as the answers were relatively short. The lack of opportunity to ask follow‐up questions to clarify the adolescents’ answers might also be a weakness. However, this applies to most questionnaires, and the choice of an open‐ended question for data collection was based on Polit and Beck 22 and Krippendorff 26, who argue it is suitable when seeking peoples’ perceptions. The transferability of the results is limited since the sample not was chosen at random. However, the result can be applied to or reflected on different situations, and thus useful for sleep‐promoting health work.

## Conclusion

To summarise the results of the present study, the adolescents’ sleeping difficulties result from an imbalance between high requirements and insufficient preconditions for a good sleep. To avoid sleeping difficulties, adolescents must address the reasons for them, such as stress, technology use, nonexistent sleeping habits, existential thoughts, needs and various forms of suffering. Adolescents may need support to find a functional balance in everyday life to deal with these reasons. While support is certainly needed from their parents, adolescents also need support from the school nurse and school health services. When supporting adolescents in finding balance, school nurses and school health services need to inspire the adolescents to good basic sleep habits and to allow themself sufficient time for sleep. All health professionals caring for adolescents should remain vigilant regarding the important issue of sleeping difficulties, as good sleep hygiene optimises adolescents’ capability for health and development.

## Conflict of interests

The authors declared no potential conflicts of interest with respect to the research, authorship and/or publication of this article.

## Author contributions

M.J. and K.H. designed the study; M.J. collected and analysed the data, in collaboration with K.H. and K.J.; M.J prepared the manuscript, in collaboration with K.J. Critical revision and supervision were provided by K.H. and K.J.

## Ethical approval

The study was approved by the Regional Ethical Review Board in Gothenburg (D. no: 588/12).

## Funding

Sparbanksstiftelsen Sjuhärad, Ebba Danelius Stiftelse, Irisstipendiet and StiftelsenTornspiran Sweden provided research funding and open access to Malin Jakobsson.
